# Interaction of gender and body mass index (BMI) reveals evidence of linkage for hypertension in the Framingham Heart Study

**DOI:** 10.1186/1471-2156-4-S1-S45

**Published:** 2003-12-31

**Authors:** Denise Daley, Shannon R Edwards, Yeunjoo Song, Dan Baechle, Sobha Puppala, JH Schick, Jane M Olson, Katrina AB Goddard

**Affiliations:** 1 Department of Epidemiology and Biostatistics, Rammelkamp Center for Education and Research, MetroHealth Campus, Case Western Reserve University, Cleveland, Ohio, USA

## Abstract

**Background:**

Genetic heterogeneity and complex biologic mechanisms of blood pressure regulation pose significant challenges to the identification of susceptibility loci influencing hypertension. Previous linkage studies have reported regions of interest, but lack consistency across studies. Incorporation of covariates, in particular the interaction between two independent risk factors (gender and BMI) greatly improved our ability to detect linkage.

**Results:**

We report a highly significant signal for linkage to chromosome 2p, a region that has been implicated in previous linkage studies, along with several suggestive linkage regions.

**Conclusion:**

We demonstrate the importance of including covariates in the linkage analysis when the phenotype is complex.

## Background

Major risk factors for cardiovascular disease (CVD) include hypertension and obesity, both of which are complex and heterogeneous. It is estimated that hypertension affects ~50 million adult Americans [1]. Although obesity [2] has been identified as a risk factor for hypertension, the exact causes of hypertension are unknown. Both conditions often coexist, suggesting that shared environmental and genetic influences may impact both traits. Population-based samples, such as the Framingham Heart Study, will include sets of individuals with different causes of disease, obscuring evidence of linkage to a particular gene if this heterogeneity is not taken into account in the linkage analysis.

We investigated hypertension as a dichotomous phenotype in the Genetic Analysis Workshop (GAW) 13 data set from the Framingham Heart Study. To allow for genetic heterogeneity, we utilized a covariate-based affected relative pair (ARP) approach [3] using obesity [measured by body mass index (BMI)], gender, and the interaction of BMI and gender as covariates, to identify candidate regions for hypertension. We show that incorporating a parameter that measures the joint action of obesity and gender on hypertension status into the linkage analysis significantly increases the linkage signal in several chromosomal regions.

## Methods

In these analyses, we utilized the phenotypic and genotypic information provided for the Framingham real data set. Individuals in Cohorts 1 and 2 were affected with hypertension if their mean for all systolic blood pressure (SBP) readings was ≥140 mm Hg or if they had at least one documented use of antihypertensive medication. Analyses conducted to check for pedigree and genotyping errors using RELTEST and MARKERINFO [4], respectively, did not reveal evidence of miss-specified relationships or non-Mendelian segregation of marker genotypes.

Three covariates were incorporated into this analysis: BMI, gender, and their interaction. BMI was calculated as the weight in pounds divided by the height squared in inches and multiplied by 703. Because there were up to 12 measurements of BMI per individual, we defined five summary BMI measurements: *first, last, mean, slope*, and *predicted*. *First *and *last *BMI represent BMI measurements at the first and last reported visits. *Mean *BMI is the average of all BMI values for each individual. The *slope *(i.e., the change in BMI over time) was the model coefficient obtained from a linear regression of age on BMI within each individual. Lastly, *predicted *BMI is the predicted value of BMI at age 50 from this linear regression. The BMI values were checked for consistency and outliers. One height measurement that was 2 feet less than the prior visit's measurement was removed. The *first, last, mean*, and *slope *BMI were adjusted for mean age and gender and *predicted *BMI was adjusted only for gender. The residuals for all BMI measures were used in subsequent linkage analyses. For all BMI measures and gender, ARP-specific covariate values were generated as the sum of the covariate values for the two individuals in the ARP minus the sample mean. The interaction term between BMI and gender was then obtained by multiplying ARP-specific values of BMI and gender for each relative pair.

We used the one-parameter conditional logistic model [3] for ARP linkage analysis. This model is implemented in the program LODPAL [4] and allows for the incorporation of covariates by fitting a single additional parameter per covariate. In terms of the offspring recurrence-risk ratio denoted as (λ_1_(*x*)), conditional on *K *covariates, *x*_*k*_, the model is parameterized as λ_1_(*x*) = exp(β + γ_k_x_k_); in terms of the recurrence-risk ratio for monozygotic twins, denoted as λ_2_(*x*), it is parameterized as λ_2_(*x*) = 3.634 λ_1_(*x) *- 2.634. The number of alleles identical by descent (IBD) was computed using multipoint algorithms incorporated into GENIBD [4]. Ten pedigrees were too large for multipoint computation using GENIBD, and were split into two or three sub-pedigrees, resulting in 341 total pedigrees. P-values were obtained for the linkage models using the asymptotic distribution of the likelihood ratio tests. In this linkage analysis we define LOD score as the likelihood ratio statistics divided by 4.605 (i.e., 2log_e_10).

We used backward selection to identify the most parsimonious model at each location with suggestive evidence for linkage defined as (*p *< 0.001); i.e., covariates were removed one at a time, starting with the gender by BMI interaction. If the interaction term could not be removed, both main effects were included in the final model. Covariates were retained in the model if the *p*-value for the likelihood ratio test was < 0.05 (i.e., the likelihood of the model with the covariate vs. the likelihood of the model without the covariate).

## Results

Of the 2898 individuals with a mean SBP reading, 1174 individuals (562 males and 612 females) were classified as having hypertension, resulting in 263 affected sib pairs (ASPs), and 450 ARPs available.

**Figure 1:**
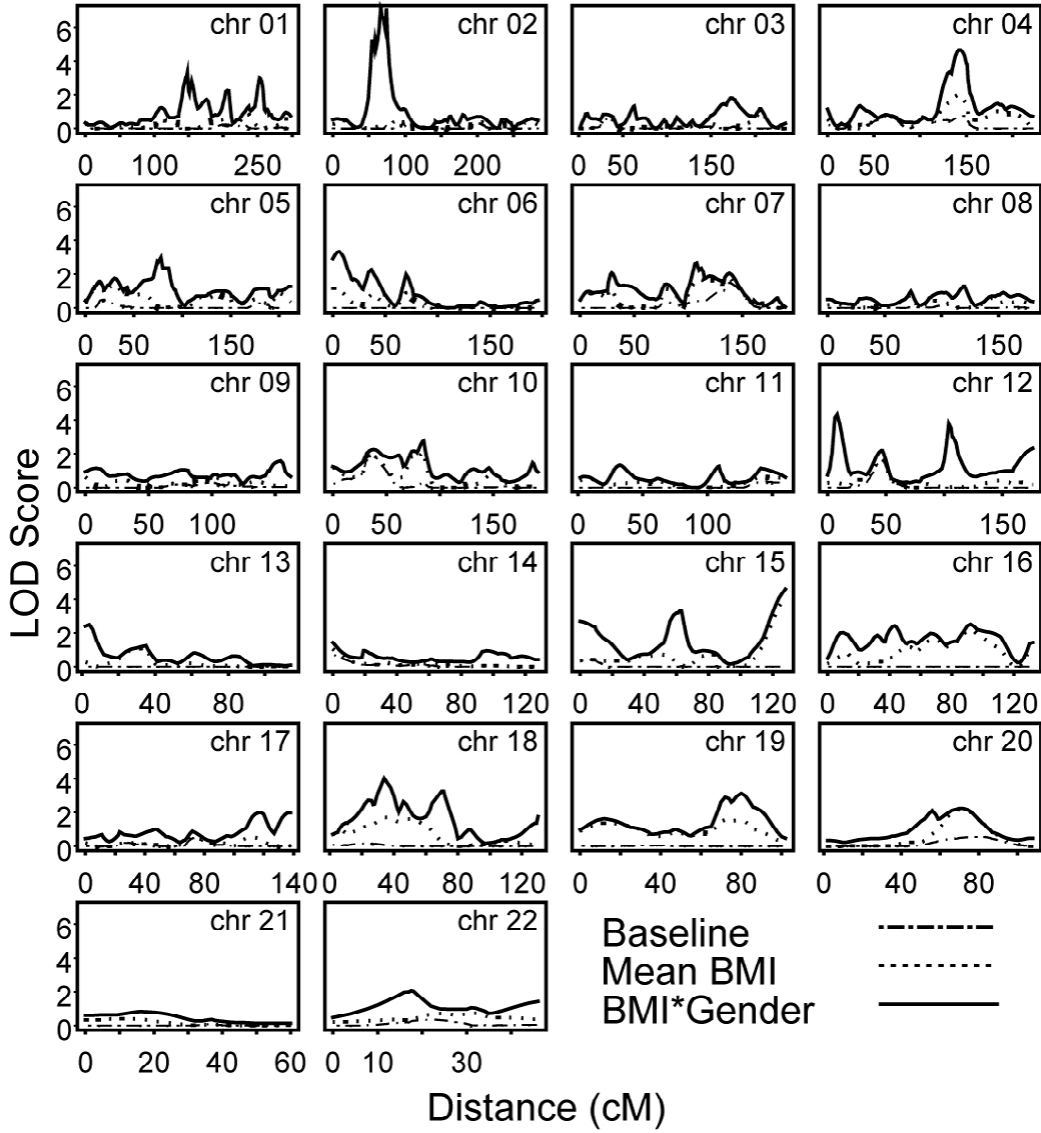
**Genome scan plot **Genome scan results for hypertension including the baseline (no covariates), the mean BMI only, and the full (mean BMI, gender, and BMI*gender interaction) models. Suggestive evidence of linkage (*p *< 0.001) corresponds to a LOD score of 2.8 or 3.8 for the single covariate and full models respectively, and significant evidence of linkage (*p *< 0.00001) corresponds to a LOD score 4.8 and 6.0, respectively.

We identified significant evidence for linkage on chromosome 2 at 74 cM, and suggestive evidence of linkage on chromosomes 1, 2 at 186 cM, 4, 12, 15, 18, 19, 20 and 22 (Table 1). Four measures of BMI (*mean*, *first*, *last*, and *predicted*) gave similar results, so we only report results for *mean *BMI. Additionally, we report results for *slope *BMI, which measures an individual's change in BMI over time. For the baseline models (without covariates), there were no regions with suggestive evidence of linkage. The highest LOD scores for the baseline models were on chromosomes 7 (LOD = 1.5, *p *= 0.004), 10 (LOD = 1.89, *p *= 0.002), and 12 (LOD = 1.68, *p *= 0.003). The only region suggestive of linkage with BMI only as the covariate was on 15q with *mean *BMI (Table 1 and Figure 1).

An interaction of BMI with gender is consistent with the observations that the prevalence and age at onset distributions differ for hypertension between the sexes, and there are reports of gender-specific effects for obesity [5], cholesterol [6], and CVD [7]. The highest LOD score for the interaction model was on chromosome 2. For this region, individuals who are male and have a high BMI have the highest sibling recurrence risk ratio (Table 2). Recurrence risks were calculated using the model coefficients in Table 1, under the assumption that both sibs were 50 years of age and had the same BMI using λ_s_(x)= 1/4 + 1/2 λ_1_(x) + 1/4 λ_2_(x). To validate the signal on chromosome 2 we also used the new Haseman-Elston regression equations as implemented in SIBPAL [4]. In contrast to the LODPAL analysis, this model utilizes discordant sib pairs in addition to the concordant sib pairs, and allows for the non-independence among different types of sib pairs. When implemented in SIBPAL [4] the interaction between BMI*gender* is highly significant (*p *= 0.009), giving further evidence to support the peak on chromosome 2 and the presence of locus heterogeneity measured by the interaction of BMI and gender.

Other regions with suggestive evidence of linkage were found on chromosomes 4 (LOD = 4.66) and 12 (LOD = 4.32). These regions consistently had high LOD scores across the four BMI measures. The most parsimonious model on chromosome 4 shows that the risk increases with BMI in both genders, but is more pronounced in males than in females, and on chromosome 12 the model suggests that males with low BMI and females with high BMI are at the greatest risk. We evaluated the recurrence risk ratio at arbitrary ages for the sibs, and found that the only region to demonstrate an effect of age was on chromosome 18, where the recurrence risk ratio increases with BMI in young males and older females.

The peak locations for BMI *slope *were different than the peaks for the other BMI measures (Table 1). When the BMI*gender interaction was included in the model, suggestive evidence of linkage was found on chromosomes 1, 2, 4, 19, 20, and 22 (Table 1). Our strongest peak on chromosome 22 is just below the cut-off for significant evidence for linkage, and the region on chromosome 19 also had suggestive evidence of linkage using the other BMI measures (LOD scores from 3.12 to 3.92).

## Conclusions

We have identified significant evidence of linkage to chromosome 2p, and suggestive evidence of linkage on chromosomes 1, 4, 12, 15, 18, 19, 20, and 22. The signal on chromosome 2p replicates the findings of five previous linkage studies including analyses of SBP in the GENOA study [8] (*p *= 0.0193), diastolic blood pressure (DBP) in the Quebec family study [9] (LOD = 1.02), and SBP in the HERITAGE study [10] (LOD = 1.88) which report linkage to D2S441 located 6 cM from our peak, and of SBP in the NHLBI Heart Study [11] (LOD = 1.1) which reports a signal at 70 cM. In addition, Hunt et al. [11] report another peak on this chromosome at 56 cM (LOD = 1.1) in hypertensive ASPs. Also the NHLBI Family Blood Pressure Program (FBPP) recently reported a LOD score of 3.09 at marker D2S1788, using BMI as a covariate and performing the analysis with LODPAL [12]. Additionally, the peak on chromosome 19 (BMI *slope*) is near the ApoE locus, which has previously been associated with risk for hypertension and CVD [7,13].

Candidate genes have not been evaluated in these regions, with the exception of chromosome 12, in which a recent case/control study, showed a significant association between the T allele of G-Protein β3-Subunit (GNB3 ~13.6 cM) and hypertensive subjects with increased BMI [14]. Functional assays demonstrate an effect of this polymorphism, with enhanced activity for the T allele [15]. Interestingly, previous linkage studies have failed to identify this region, including that of Rice et al. [9], who typed markers located within GNB3.

Our results differ substantially from the results of the previous genome scan in the Framingham Heart Study population that utilized SBP as a quantitative trait [16]. We did not identify the region on chromosome 17 reported by Levy et al. [16], nor were the linkage signals detected in this analysis identified in that study. Genes impacting the normal variation in blood pressure (measured by SBP as a quantitative trait) may be very distinct from genes impacting extreme hypertension (measured as a discrete trait).

Because *slope *BMI measured the individual change in BMI over time, this covariate may have identified different signals associated with the normal variation in blood pressure. It is interesting to note that the recurrence risk ratios for *slope *BMI consistently (four out of six signals) showed increasing risk for women with positive slopes, or women who gain weight over time, while the other measures of BMI did not show consistent linkage to either men or women. Furthermore, it should be noted that we used a linear regression equation to model slope BMI; however, during the GAW13 meeting Strug et al. [17] demonstrated that *slope *BMI did not fit a linear model, but was characterized by a gain phase lasting to approximately age 55 followed by a less extreme decreasing phase in older age, and our findings may change if we were to model *slope *BMI in this fashion.

We demonstrate the importance of including covariates in linkage analyses that may account for locus heterogeneity. The significant linkage signals on chromosome 2, and most of our other candidate regions, were not detected without including the gender*BMI interaction term. The signal on chromosome 2 is stronger in our analysis than in previous reports and is not accounted for by differences in sample size. It is noteworthy that we are able to detect these signals despite using a broad definition of hypertension. The models developed in our analysis identify phenotypic characteristics of individuals likely to be linked to each candidate region and suggest that complex traits such as hypertension may have gender-specific components. Our results show that some loci influencing hypertension may have multiple additional interacting factors, revealing the complexity of the disease. This information facilitates the identification of target candidate genes and molecular pathways for each region, and also aids in the identification of appropriate populations for confirmatory studies.

## Figures and Tables

**Table 1:** Regions with at least suggestive linkage (*p *< 0.001) for the most parsimonious model

Chromosome (Location in cM)	Nearest Marker	Lod Score (p-value)		Model Coefficients			
			
		*Mean *BMI	*Slope *BMI	β	γ_GENDER_	γ_BMI_	γ_GENDER*BMI_
2 (74 cM)	D2S1352	7.1 (8.76 × 10^-7^)		0.000	-0.070	0.035	-0.059
4 (142 cM)	D4S1644	4.7 (0.00017)		0.330	0.027	0.062	-0.052
12 (8 cM)	D12S372	4.3 (0.00035)		0.030	-0.171	0.012	0.050
15 (128 cM)	D15S642	4.1^A^(0.00004)		0.041		-0.041	
18 (34 cM)	D18S843	4.0 (0.00068)		0.229	-0.175	0.069	-0.050
1 (276 cM)	D1S547		3.9 (0.00091)	0.087	-0.176	0.621	0.687
2 (186 cM)	D2S1384		4.3 (0.00037)	0.000	-0.077	0.151	1.205
4 (194 cM)	D4S2417		4.1 (0.00057)	0.000	-0.002	0.571	-1.134
19 (75 cM)	D19S589		4.3 (0.00037)	0.018	-0.218	0.504	0.577
20 (74 cM)	D20S481		4.0 (0.00072)	0.146	0.066	-0.783	-0.713
22 (48 cM)	D22S445		5.7 (0.00001)	0.157	0.311	0.594	1.196

^A^LOD score for a model that includes BMI only. Otherwise, LOD scores are for the full model.

**Table 2:** Sibling recurrence risk ratios on chromosome 2 (74 cM)

	λ_s_^A^		
	
*Mean *BMI	Male/Male	Male/Female	Female/Female
25	1.41	0.43	0.77
30	1.76	1.27	0.72
35	5.21	2.00	0.48

^A^λ_s _computed for the same *mean *BMI and age (50 yr) for each individual.

## References

[B1] Burt VL, Whelton P, Roccella EJ, Brown C, Cutler JA, Higgins M, Horan MJ, Labarthe D (1995). Prevalence of hypertension in the US adult population. Results from the Third National Health and Nutrition Examination Survey, 1988-1991. Hypertension.

[B2] MacMahon SW, Blacket RB, Macdonald GJ, Hall W (1984). Obesity, alcohol consumption and blood pressure in Australian men and women. The National Heart Foundation of Australia Risk Factor Prevalence Study. J Hypertens.

[B3] Goddard KAB, Witte JA, Suarez BK, Catalona WJ, Olson JM (2001). Model-free linkage analysis with covariates confirms linkage of prostate cancer to chromosomes 1 and 4. Am J Hum Genet.

[B4] Case Western Reserve University (2002). S.A.G.E. Statistical Analysis for Genetic Epidemiology, Release 4.2. Cleveland, OH, Department of Epidemiology and Biostatistics, Case Western Reserve University.

[B5] Stone S, Abkevich V, Hunt SC, Gutin A, Russell DL, Neff CD, Riley R, Frech GC, Hensel CH, Jammulapati S, Potter J, Sexton D, Tran T, Gibbs D, Iliev D, Gress R, Bloomquist B, Amatruda J, Rae PM, Adams TD, Skolnick MH, Shattuck D (2002). A major predisposition locus for severe obesity at 4p15-p14. Am J Hum Genet.

[B6] Seman LJ, DeLuca C, Jenner JL, Cupples LA, McNamara JR, Wilson PVF, Castelli WP, Ordovas JM, Schaefer EJ (1999). Lipoprotein (a)-cholesterol and coronary heart disease in the Framingham Heart Study. Clin Chem.

[B7] Lahoz C, Schaefer EJ, Cupples A, Wilson PVF, Levy D, Osgood D, Parpos S, Pedro-Botet J, Daly JA, Ordovas JM (2001). Apolipoprotein E genotype and cardiovascular disease in the Framingham Heart Study. Atherosclerosis.

[B8] Krushkal J, Ferrell R, Mockrin SC, Turner ST, Sing CF, Boerwinkle E (1999). Genome-wide linkage analyses of systolic blood pressure using highly discordant siblings. Circulation.

[B9] Rice T, Rankinen T, Province MA, Chagnon YC, Perusse L, Borecki IF, Bouchard C (2000). Genome-wide analysis of systolic and diastolic blood pressure the Quebec Family Study. Circulation.

[B10] Rice T, Rankinen T, Chagon YC, Province MA, Perusse L, Leon AS, Skinner JS, Wilmore JH, Bouchard C, Rao DC (2002). Genomewide linkage scan of resting blood pressure HERITAGE Family Study. Hypertension.

[B11] Hunt SC, Ellison RC, Atwood LD, Pankow JS, Province MA, Leppert MF (2002). Genome scans for blood pressure and hypertension: the National Heart, Lung, and Blood Institute Family Heart Study. Hypertension.

[B12] Wu X, Thiel B, Kardia S, Rao DC, Province M, Ranade K, Zhu X, Luke A, Kan D, Cooper R (2002). Affected-relative-pair linkage analysis with covariates for hypertension in NHLBI Family Blood Pressure Program (FBPP). Am J Hum Genet.

[B13] Allayee H, deBruin TW, Dominguez KM, Cheng LS, Ipp E, Cantor RM, Krass KL, Keulen ETP, Aouizerat BE, Lusis AJ, Rotter JI (2001). Genome scan for blood pressure in Dutch dyslipidemic families reveals linkage to a locus on chromosome 4p. Hypertension.

[B14] Siffert W, Rosskopf D, Siffert G, Busch S, Moritz A, Erbel R, Sharma AM, Ritz E, Wichmann HE, Jakobs KH, Horsthemke B (1998). Association of a human G-protein β3 subunit variant with hypertension. Nat Genet.

[B15] Pietruck F, Moriz A, Montemurro M, Sell A, Busch S, Rosskopf D, Virchow S, Esche H, Brockmeyer N, Jakobs KH, Siffert W (1996). Selectively enhanced cellular signaling by G_i _proteins in essential hypertension. Circ Res.

[B16] Levy D, DeStefano AL, Larson MG, O'Donnell CJ, Lifton RP, Gavras H, Cupples LA, Myers RH (2000). Evidence for a genome influencing blood pressure on chromosome 17. Genome scan linkage results for longitudinal blood pressure phenotypes in subjects from the Framingham Heart Study. Hypertension.

[B17] Strug L, Sun L, Corey M (2003). The genetics of cross-sectional and longitudinal BMI. BMC Genetics.

